# HPV16 E6-E7 induces cancer stem-like cells phenotypes in esophageal squamous cell carcinoma through the activation of PI3K/Akt signaling pathway *in vitro* and *in vivo*

**DOI:** 10.18632/oncotarget.10959

**Published:** 2016-07-30

**Authors:** Ruxing Xi, Shupei Pan, Xin Chen, Beina Hui, Li Zhang, Shenbo Fu, Xiaolong Li, Xuanwei Zhang, Tuotuo Gong, Jia Guo, Xiaozhi Zhang, Shaomin Che

**Affiliations:** ^1^ Department of Radiotherapy, The First Hospital Affiliated of Xi'an Jiao Tong University, Xi'an, Shaan Xi, 710061, P.R.China; ^2^ Department of Radiotherapy, People's Hospital of Shaanxi Province, Xi'an, Shaan Xi, 710068, P.R.China; ^3^ Department of Radiotherapy, The People's Liberation Army 323 Hospital, Xi'an, Shaan Xi, 710054, P.R.China

**Keywords:** esophageal squamous cell carcinoma, HPV16 E6-E7, CSCs, PI3K, p75NTR

## Abstract

High-risk human papillomavirus (HPV), especially HPV16, correlates with cancerogenesis of human esophageal squamous cell carcinoma (ESCC) and we have reported that HPV16 related with a poor prognosis of ESCC patients in China. We aim to investigate the potential role and mechanism of HPV16 in ESCC development and progress. Our following researches demonstrated that ESCC cells which were stably transfected by HPV16 E6-E7 lentiviral vector showed a remarkable cancer stem-like cells (CSCs) phenotype, such as: migration, invasion, spherogenesis, high expression of CSCs marker in ESCC---p75NTR, chemoresistance, radioresistance, anti-apoptosis ability *in vitro* and cancerogenesis *in vivo*. HPV16 E6-E7 induced PI3K/Akt signaling pathway activation and this affect could be effectively inhibited by LY294002, a specific PI3K inhibitor. It was also indicated that the inhibition of PI3K/Akt signaling pathway by PI3K and Akt siRNA reverse the effect which induced by HPV16 E6-E7 in ESCC cells. Taken together, the present study demonstrates that HPV16 E6-E7 promotes CSCs phenotype in ESCC cells through the activation of PI3K/Akt signaling pathway. Targeting the PI3K/Akt signaling pathway in HPV16 positive tissues is an available therapeutic for ESCC patients.

## INTRODUCTION

Esophageal carcinoma was the sixth and ninth lethal malignancy worldwide in men and women in 2012 [1], respectively. Although the advanced treatment such as radical surgical resection, chemotherapy and radiotherapy are extensively applied, the 5-year survival rate of esophageal squamous cell carcinoma (ESCC) patients remained less than 30%, resulting in 400,200 deaths in 2012 worldwide [2].

The etiology of ESCC has not been fully elucidated although epidemiological and experimental studies have identified several risk factors [1, 3, 4]. Human papillomavirus (HPV) infection in ESCC was first suggested as one of the risk factors in 1982 [5] and has been detected in ESCC in high-risk areas in China recently [6]. High-risk HPV, including HPV16 and HPV18, expresses oncogenes E6 and E7 which can be bound to the p53 and Rb tumor suppressor, respectively, leading the rapid p53 degradation and the loss of Rb products. This might suggest the etiological involvement of high-risk HPV in ESCC [7–10].

Cancer stem-like cells (CSCs) are a small subpopulation of tumor cells with the capacity of self-renewal maintaining tumor growth and cell differentiation. CSCs also contribute to the tumorigenic potential of cancer, including spherogenesis, resistance to cytotoxic drugs and ionizing radiation [11–13]. The low-affinity p75 neurotrophin receptor (p75NTR) was first identified as a stem/progenitor cell marker in human normal esophageal epithelial cells by Okumura in 2003 [14]. Moreover, p75NTR is necessary for survival and maintenance of ESCC tumors and could be considered as a potential target for novel therapies [15]. The p75NTR positive cells are supposed to be capable of repopulating all known epithelial cell subtypes with a slow cycling rate and a relatively immature phenotype. It could be concluded that p75NTR could be regarded as a candidate biomarker of CSCs in ESCC cells.

Activation of PI3K/Akt signaling pathway was required for colony-formation ability *in vitro* and tumorigenicity *in vivo* in breast CSCs [16]. It was also found that targeting PI3K benefits for prostate cancer by eliminate prostate CSCs [17, 18]. Up-regulation of hypoxia-inducible factor 1α inhibits proliferation and promotes survival of prostate cancer stem cells through the PI3K [19]. Furthermore, PI3K/Akt inhibition is considered to be an effective strategy in the solid-tumor treatment which reduced the bulk tumor burden significantly combined with classical chemotherapy [20].

Our previous study demonstrated that HPV16 infection and highly PI3K expression in ESCC patients were independently associated with poor overall survival [21]. Therefore, a better understanding of ESCC biology with high-risk HPV infection is urgently needed to be detected. In the present study, we first detected the influence and the potential mechanism of HPV16 E6-E7 expression in ESCC cells *in vitro* and *in vivo*. LY294002, a specific PI3K inhibitor suppressing PI3K/Akt pathways which plays a vital role in carcinogenesis, was selected due to its small size and ease of use *in vitro* and *in vivo* systems. The results demonstrated that HPV16 E6-E7 expression promoted CSCs phenotypes in ESCC cells *via* activating PI3K/Akt signaling pathway *in vitro* and *in vivo*.

## RESULTS

### Lentivirus transfection of ESCC cells and confirmation of HPV16 E6-E7 expression

**Figure 1:**
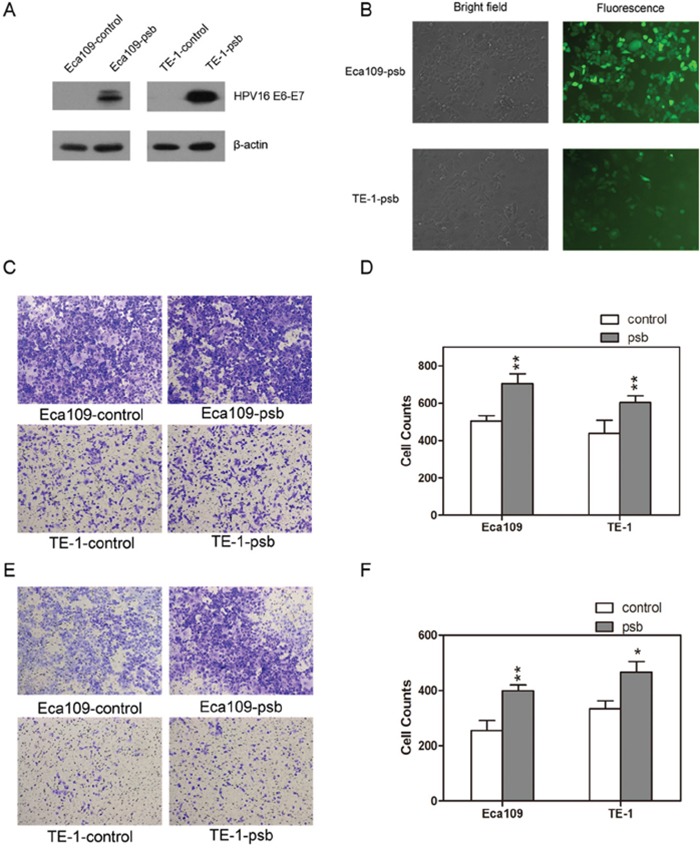
HPV16 E6-E7 lentiviral vector stably transfected ESCC cells **A.** Western blotting detected the HPV16 E6-E7 protein expression in ESCC cells (Eca109-control, Eca109-psb, TE-1-control and TE-1-psb cells). **B.** Transfection outcome of HPV16 E6-E7 lentiviral vector in Eca109-psb and TE-1-psb cells were observed under fluorescence microscope after 72h. Transwell assay was performed to evaluate the migration **C.** and invasion **E.** ability between Eca109-control and Eca109-psb cells, TE-1-control and TE-1-psb cells. Migration **D.** and invasion **F.** results were quantified as histograms. Data are represented as mean±S.D. of three independent experiments. *P<0.05, **P<0.01.

HPV16 E6-E7 protein was stably expressed in HPV16 E6-E7 lentiviral vector transfected ESCC cells, which were labeled as Eca109-psb and TE-1-psb cells, while no expression of HPV16 E6-E7 protein was observed in control vector transfected ESCC cells, which were labeled as Eca109-control and TE-1-control cells. The transfection efficiency were detected and confirmed by western blotting (Figure 1A). The transfection outcome of HPV16 E6-E7 lentiviral vector was observed using a fluorescence microscope 72 h after experimental treatment of Eca109 and TE-1 cells (Figure 1B).

### HPV16 E6-E7 promotes CSCs phenotypes, increases the ratio of CSCs marker-p75NTR, and reduces G_0_/G_1_ cell cycle arrest in ESCC cells *in vitro*

The migration and invasion capacities of ESCC cells were conducted by the transwell assay, as results were shown (Figure 1C and 1E) and quantified (Figure 1D and 1F). Relative to control cells, there were dramatically (*P*<0.05-0.01) improvements in the migration capacity or invasion capacity of Eca109-psb and TE-1-psb cells after 24h (without matrigel) migration assay or 48h (with matrigel) invasion assay, respectively. It could be concluded that HPV16 E6-E7 increases migration and invasion potential in ESCC cells *in vitro*.

**Figure 2:**
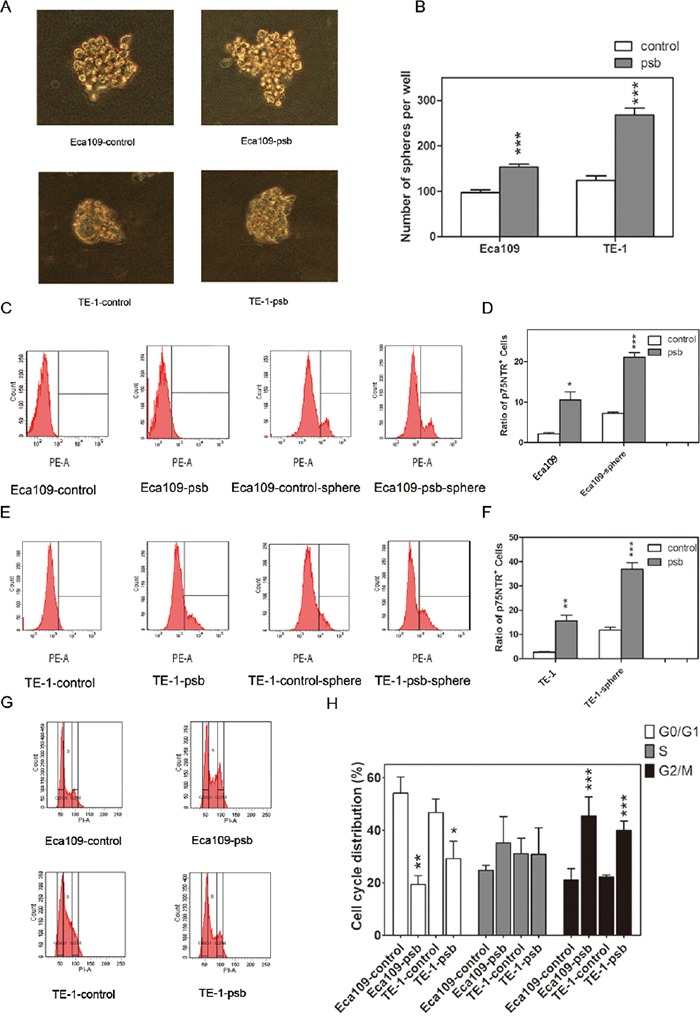
HPV16 E6-E7 promotes spherogenesis ability and reduces G0/G1 cell cycle arrest in ESCC cells **A.** Representative images of spheres (diameter≥75μm) formed from Eca109-control, Eca109-psb, TE-1-control and TE-1-psb cells after 14 days of culture in SFM. **B.** Spheres were counted under microscope and quantified as histograms. **C** and **E.** The p75NTR positive cells in ESCC cells and spheres formed from ESCC cells were measured by flow cytometry. Ratio of p75NTR positive cells in each cells were quantified in **D** and **F.** as histograms. **G.** DNA content analysis was operated by flow cytometry to reflect the cell cycle distribution of ESCC cells. Results were quantified as histograms in **H.** Data are represented as mean±S.D. of three independent experiments. *P<0.05, **P<0.01,***P<0.001. Eca109-control and TE-1-control compared with the Eca109-psb and TE-1-psb, respectively.

The studies above demonstrated that HPV16 E6-E7 behaved as a tumor-promoting factor in ESCC cells. We next examined the effect of HPV16 E6-E7 on the spherogenesis ability of ESCC cells which was considered to be an important characteristic of CSCs *in vitro* [22–24]. It was observed obviously (*P*<0.001) increased in the number of spheres in Eca109-psb and TE-1-psb cells compared to control cells on day 14(Figure 2A and 2B). It shows that HPV16 E6-E7 enhances the spherogenesis of ESCC cells *in vitro*.

Previous studies [14, 15] reported that the p75NTR could be a candidate CSCs marker in ESCC cells. In this study, flow cytometry was applied to investigate whether HPV16 E6-E7 increased p75NTR positive cells in ESCC cells, as it increased sphereogenesis as demonstrated above. As shown in Figure 2C-2F, significant (*P*<0.05-0.001) improvement of p75NTR positive cells were detected in Eca109-psb and TE-1-psb cells than control cells. Similarly, it was also found that the p75NTR positive cells significantly (*P*<0.01-0.001) increased in Eca109-psb and TE-1-psb cell spheres compared to control cell spheres. This suggests that HPV16 E6-E7 increases the ratio of p75NTR positive cells in ESCC cells *in vitro*.

Flow cytometry was performed to investigate the effect of HPV16 E6-E7 on ESCC cells cycle progression. As shown in Figure 2G and 2H, compared to control cells, HPV16 E6-E7 significantly (*P*<0.05-0.01) decreased cells that were arrested at the G0/G1 transition evidenced by a less accumulated cells in the G0/G1 peak. A marked (*P*<0.001) accumulation of cells at the G2/M phase was also observed. This indicates that HPV16 E6-E7 leads to the accumulation of cells in G2/M and extensively decreases the percentage of cells in G0/G1 phase in ESCC cells *in vitro*.

### HPV16 E6-E7 increases chemoresistance, radioresistance and anti-apoptosis ability after ionizing radiation in ESCC cells *in vitro*

**Figure 3:**
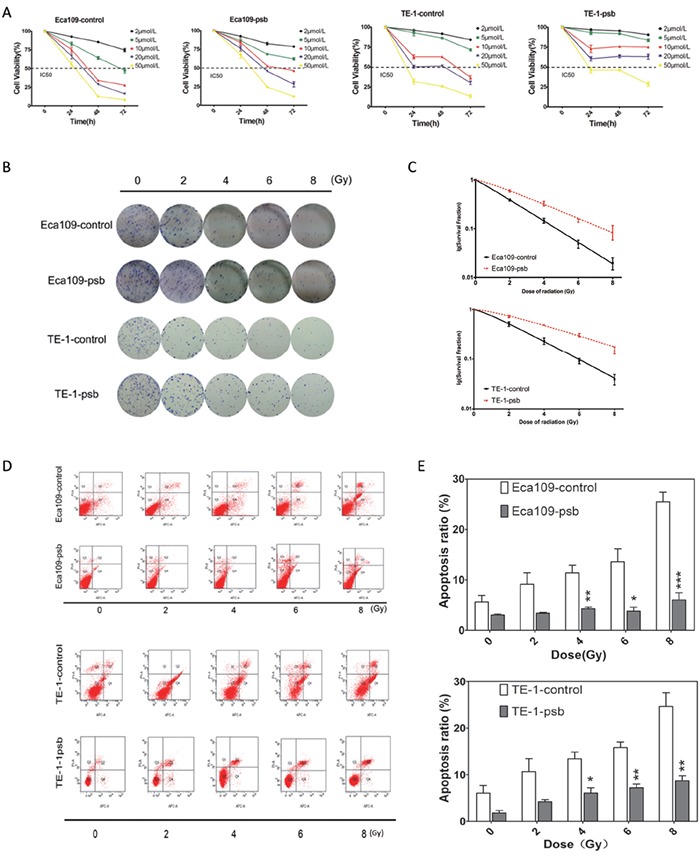
HPV16 E6-E7 increases chemoresistance, radioresistance and anti-apoptosis ability after ionizing radiation in ESCC cells **A.** Time-dependent inhibition of CDDP in ESCC cells were evaluated by CCK8 assay. **B.** Colony formation assay of ESCC cells. **C.** Dose-survival curve of ESCC cells were drawn based on the data of colony formation assay. **D.** Cell apoptosis after ionizing radiation of ESCC cells were analysed by Annexin V-APC/PI flow cytometry and quantified as histograms in. **E.** All the data above are represented as mean±S.D. of three independent experiments. *P<0.05, **P<0.01,***P<0.001.

CCK-8 assay was performed to determine whether HPV16 E6-E7 had an effect on ESCC cell proliferation after cisplatinum (CDDP), one of the representative cytotoxic drugs, treatment. As shown in Figure 3A, cells were treated with 2, 5, 10, 20, 50μmol/L CDDP. The 50% growth inhibitory concentrations (IC_50_) of CDDP for Eca109-control, Eca109-psb, TE-1-control and TE-1-psb cells at 48h were 7.615, 13.41, 19.44 and 39.31 μmol/L, respectively, at 72h were 4.704, 8.079, 8.613, and 26.27μmol/L, respectively. It could be obviously concluded that all of the IC50 value of Eca109-psb and TE-1-psb cells at 48 and 72h are significantly (*P*<0.001) higher than control cells. These results above indicate that HPV16 E6-E7 increases chemoresistance in ESCC cells *in vitro*.

The colony formation assay was performed to determine the influence of HPV16 E6-E7 on radioresistance in ESCC cells. Cells were divided and exposed to 0, 2, 4, 6, 8 Gy of ionizing radiation (Figure 3B). The Single hit multi-target model SF=1-(1-e^−D/D0^)^n^ [25] was applied to fitting the result and dose-survival curves of ESCC cells (Figure 3C). Constant values of radiation biology were calculated basing on the data of colony formation assay (Supplementary Table S1). It was found that the value of D_0_, k and SF_2_ significantly (*P*<0.001-0.05) increased in Eca109-psb and TE-1-psb cells compared to control cells (Supplementary Table S1) while no significant differences were observed in the value of N, Dq. This indicates that HPV16 E6-E7 also increases radioresistance of ESCC cells *in vitro*.

Annexin V-APC/PI flow cytometry was applied to assess the effect of HPV16 E6-E7 on ESCC cells apoptosis after ionizing radiation. The fourth quadrant (Q4) represents early apoptosis cells and the second quadrant (Q2) represents late apoptotic and necrotic cells (Figure 3D). The ratio of apoptosis cells increased with radiation dose accumulating in all the cells (Figure 3E). Meanwhile, the ratio of apoptosis cells was significantly (*P*<0.05-0.001) lower in Eca109-psb and TE-1-psb cells than control cells when treated by 4, 6 and 8Gy (Figure 3E), with no significant differences by 2Gy (*P*=0.065, 0.087, respectively) or untreated (*P*=0.118, 0.069, respectively). This means that HPV16 E6-E7 inhibits the apoptosis in ESCC cells when treated by ionizing radiation, especially accumulating and highly dose of ionizing radiation *in vitro*.

### HPV16 E6-E7 increased p75NTR expression through PI3K/Akt signaling pathway in ESCC cells

As described above, more and more recent studies show that PI3K/Akt pathway plays a vital role in the maintenance of CSCs [26–28]. So, we speculate that HPV16 E6-E7 may increase the CSCs properties of ESCC cells through up-regulation of PI3K/Akt signaling.

**Figure 4:**
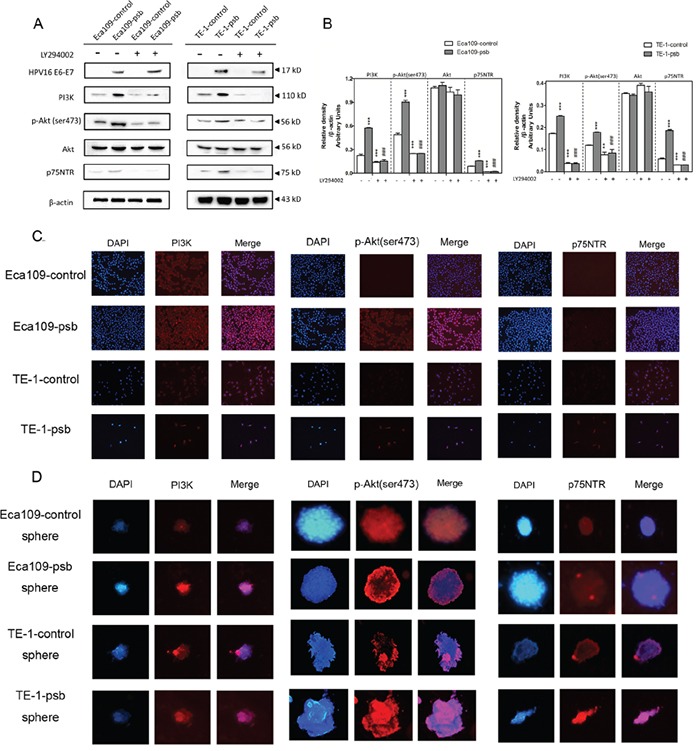
PI3K/Akt signaling pathway activation and increased p75NTR expression induced by HPV16 E6-E7 were inhibited by LY294002 in ESCC cells **A.** Proteins involved in PI3K/Akt signaling pathway and p75NTR protein were analysed by western blotting. Cells were treated with LY294002 (10μmol/L) for 24h. Equal protein loading was evaluated by β-actin. **B.** Densitometric of western blotting bands in Figure A were analyzed and expressed relative to the loading control, β-actin. Data are typical of three experiments and the histogram values are mean ± S.D. **P<0.01,***P<0.001, relative to control cells without LY294002 treatment. ###*P*<0.001, relative to psb cells without LY294002 treatment. **C.** p-Akt, PI3K and p75NTR proteins of ESCC cells were detected by immunofluorescence. **D.** PI3K and p75NTR proteins of spheres formed from ESCC cells were detected by immunofluorescence.

Western blotting and immunofluorescence were applied to examine the hypothesis above. The western results were shown in Figure 4A and relative density were analyzed and quantified as histograms in Figure 4B. The expression of PI3K, phosphorylation-Akt (ser473), also named as p-Akt (ser473), and p75NTR in the Eca109-psb and TE-1-psb cells significantly (*P*<0.001) increased compared to control cells. Whereas, no significant alterations could be observed in the expression of total Akt in Eca109-psb and TE-1-psb cells compared to control cells (Figure 4A and 4B). Immunofluorescence of ESCC cells (Figure 4C) demonstrated that PI3K, p-Akt (ser473) and p75NTR expressions were markedly increased in Eca109-psb and TE-1-psb cells compared to control cells. Meanwhile, immunofluorescence of spheres (Figure 4D) also demonstrated that PI3K, p-Akt (ser473) and p75NTR expression are obviously stronger in spheres derived from Eca109-psb and TE-1-psb cells than spheres derived from control cells. It suggests that HPV16 E6-E7 activates the PI3K/Akt signaling pathway in ESCC cells and spheres derived from ESCC cells.

To investigate whether inhibiting the PI3K/Akt signaling pathway can reverse CSCs phenotypes caused by HPV16 E6-E7. LY294002 was applied at the concentration of 10μmol/L in the cell culture medium incubating for 24h as our previously study described [29]. Consequently, there were no significant difference in the expression of PI3K, p-Akt (ser473) and p75NTR as they all decreased (*P*<0.001) in ESCC cells after LY294002 treatment (Figure 4A and 4B). However, there were still no alternations in the expression of total Akt in the ESCC cells after being treated with LY294002 (Figure 4A and 4B).

Based on the results above, LY294002 treatment reverse the expression of PI3K, p-Akt (ser473) and p75NTR in Eca109-psb and TE-1-psb cells, and this further demonstrated that the activation of PI3K/Akt signaling pathway, induced by HPV16 E6-E7, plays a vital role in maintaining CSCs phenotypes in ESCC cells [30].

**Figure 5:**
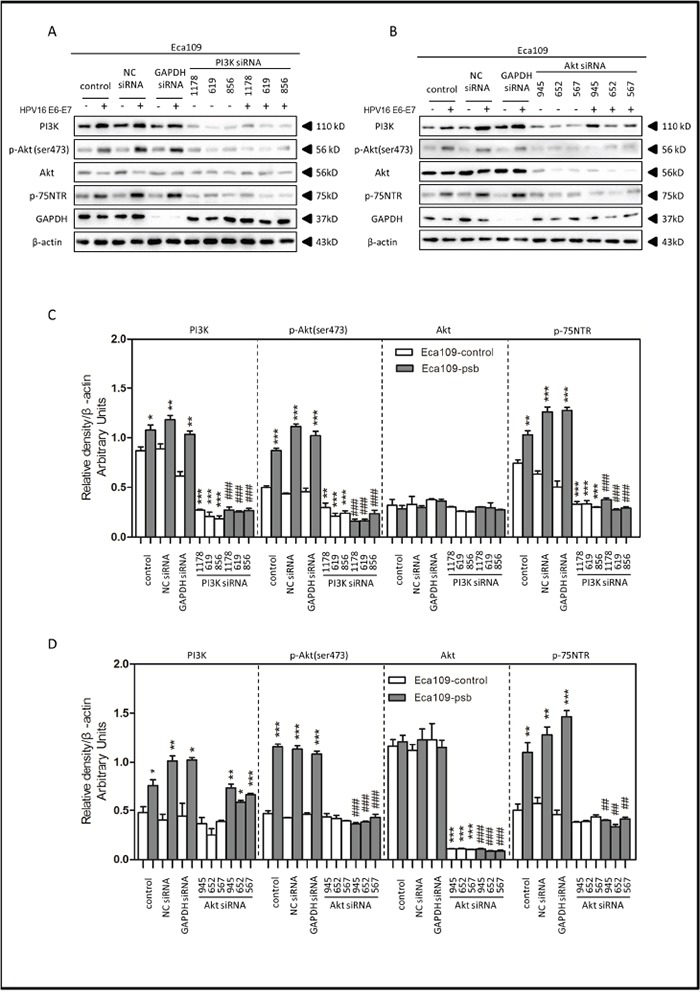
HPV16 E6-E7 increased p75NTR expression through PI3K/Akt signaling pathway in Eca109 cells **A.** Proteins involved in PI3K/Akt signaling pathway and p75NTR protein were analysed by western blotting. Eca109 cells were treated by negative control RNA, GAPDH RNA, PI3K siRNA, respectively. Equal protein loading was evaluated by β-actin. “-” means HPV16 E6-E7 negative which represents Eca109-control cells, “+”means HPV16 E6-E7 positive which represents Eca109-psb cells. **B.** Proteins involved in PI3K/Akt signaling pathway and p75NTR protein were analysed by western blotting. Eca109 cells were treated by negative control RNA, GAPDH RNA, Akt siRNA, respectively. Equal protein loading was evaluated by β-actin. “-”means HPV16 E6-E7 negative which represents Eca109-control cells, “+”means HPV16 E6-E7 positive which represents Eca109-psb cells. **C.** Densitometric of western blotting bands in Figure A were analyzed and expressed relative to the loading control, β-actin. Data are typical of three experiments and the histogram values are mean ± S.D. *P<0.05,**P<0.01,***P<0.001, relative to control cells in control siRNA group. ###*P*<0.001, relative to Eca109-psb cell in control siRNA group. **D.** Densitometric of western blotting bands in Figure B were analyzed and expressed relative to the loading control, β-actin. Data are typical of three experiments and the histogram values are mean ± S.D. *P<0.05,**P<0.01,***P<0.001, relative to control cells in control siRNA group or Akt siRNA group. ###*P*<0.001, relative to Eca109-psb cell in control siRNA group.

**Figure 6:**
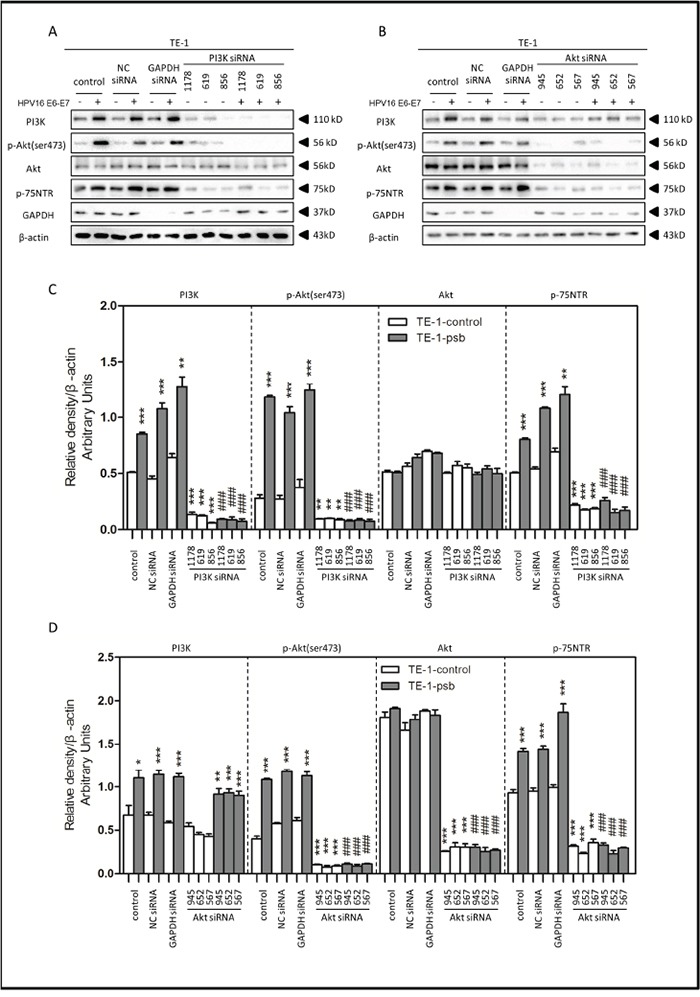
HPV16 E6-E7 increased p75NTR expression through PI3K/Akt signaling pathway in TE-1 cells **A.** Proteins involved in PI3K/Akt signaling pathway and p75NTR protein were analysed by western blotting. TE-1 cells were treated by negative control RNA, GAPDH RNA, PI3K siRNA, respectively. Equal protein loading was evaluated by β-actin. “-” means HPV16 E6-E7 negative which represents TE-1-control cells, “+” means HPV16 E6-E7 positive which represents TE-1-psb cells. **B.** Proteins involved in PI3K/Akt signaling pathway and p75NTR protein were analysed by western blotting. TE-1 cells were treated by negative control RNA, GAPDH RNA, Akt siRNA, respectively. Equal protein loading was evaluated by β-actin. “-” means HPV16 E6-E7 negative which represents TE-1-control cells, “+” means HPV16 E6-E7 positive which represents TE-1-psb cells. **C.** Densitometric of western blotting bands in Figure A were analyzed and expressed relative to the loading control, β-actin. Data are typical of three experiments and the histogram values are mean ± S.D. *P<0.05,**P<0.01,***P<0.001, relative to control cells in control siRNA group. ###*P*<0.001, relative to TE-1-psb cell in control siRNA group. **D.** Densitometric of western blotting bands in Figure B were analyzed and expressed relative to the loading control, β-actin. Data are typical of three experiments and the histogram values are mean ± S.D. *P<0.05,**P<0.01,***P<0.001, relative to control cells in control siRNA group or Akt siRNA group. ###*P*<0.001, relative to TE-1-psb cell in control siRNA group.

Specific siRNAs were utilized to inhibit PI3K/Akt pathway for further investigation. Negative control siRNA (NC siRNA) and GAPDH siRNA were both utilized for negative and positive control. Results were shown and relative density were analyzed and quantified as histograms in Figure 5 and Figure 6.

Treated with the PI3K siRNA 1178, 619 and 856, the expression of PI3K, p-Akt (ser473) and p75NTR in ESCC cells were all significantly (*P*<0.01-0.001) decreased in siRNA group, compared to control siRNA group, (Figure 5A, 5C and Figure 6A, 6C), but no significant alternations were observed in total Akt expressions (Figure 5A, 5C and Figure 6A, 6C). Consistently, the PI3K, p-Akt (ser473) and p75NTR expressions were significantly higher (*P*<0.05-0.001) in Eca109-psb and TE-1-psb cells than control cells in control siRNA groups (Figure 5A, 5C and Figure 6A, 6C) with no significant difference of that in the PI3K siRNA groups (Figure 5A, 5C and Figure 6A, 6C).

Akt siRNA 945, 652 and 567 were utilized for Akt inhibition. No alterations of PI3K expressions were observed in Akt siRNA group, compared to control siRNA group in ESCC cells (Figure 5B, 5D and Figure 6B, 6D). Total Akt expressions significantly (*P*<0.001) decreased in Akt siRNA group, compared to control siRNA group in ESCC cells (Figure 5B, 5D and Figure 6B, 6D). The p-Akt (ser473) and p75NTR expressions significantly (*P*<0.001) decreased in Akt siRNA group, compared to control siRNA group, in Eca109-psb and TE-1-control, TE-1-psb cells (Figure 5B, 5D and Figure 6B, 6D). Inconsistently, no alternations of p-Akt (ser473) expression and p75NTR expression were observed between control siRNA group and Akt siRNA group in Eca109-control cells (Figure 5B, 5D and Figure 6B, 6D).

Interestingly, the PI3K expressions were significantly (*P*<0.05-0.001) higher in Eca109-psb and TE-1-psb, compared to control cells in control siRNA group and Akt siRNA group (Figure 5B, 5D and Figure 6B, 6D). p-Akt (ser473) and p75NTR expressions were significantly (*P*<0.05-0.001) higher in Eca109-psb and TE-1-psb cells, compared to control cells, in control siRNA group with no significant difference of that in the Akt siRNA group. GAPDH was used for positive control to make sure siRNA knock-down efficiency and β-actin was used as the endogenous reference.

### HPV16 E6-E7 promotes the tumorigenesis and radioresistance of ESCC cells *in vivo* which could be inhibited by LY294002

The xenograft nude mouse model was established to verify the effects of HPV16 E6-E7 on tumorigenesis in ESCC cells *in vivo*. The mice were randomly distributed into four groups (N=6): Control, Radiation, LY294002 and LY294002+Radiation group.

**Figure 7:**
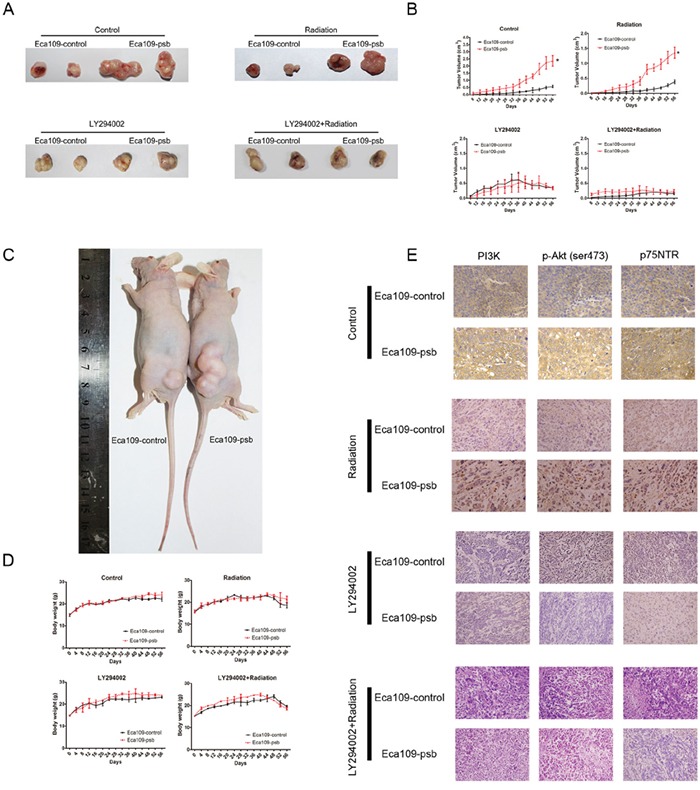
HPV16 E6-E7 promotes ESCC cells progress and radioresistance *in vivo* which are blocked by LY294002 via PI3K/Akt pathway **A.** Representative tumor pictures of control, radiation, LY294002, LY294002+radiation groups are shown. **B.** Tumor volume was recorded every 4 days from mice were employed in the study after 8 days and evaluated on the 56 days. **C.** Representative pictures of xenograft nude model. **D.** Body weight was recorded every 4 days from mice were employed in the study until 56 days. **E.** Immunohistochemical staining of tumor specimens were captured for the detection of the PI3K, p-Akt and p75NTR expression *in vivo*. Data and pictures are represented as mean ± S.D. of 6 mice in each group. *P<0.05.

The xenograft tumour growth markedly (*P*<0.05) increased in mice bearing Eca109-psb cells, compared to the Eca109-control cells, both in Control group and Radiation group (Figure 7A, 7B). However, there was no significant difference in the tumor volumes between LY294002 group and the LY294002+radiation group. Moreover, no marked changes in body weight were found in the four groups while the growth of body weight in LY294002+radiation group is the slowest in four groups (Figure 7D). This indicates that tumors derived from Eca109-psb cells grow markedly and significantly (*P*<0.05) faster than tumors derived from control cells in Control group and Radiation group. Interestingly, the significant difference could be eliminated when intraperitoneally injected with LY294002.

After tumors were surgically excised and fixed, IHC were performed to detect the mechanism involved *in vivo*. Consistent with the results of the western blotting *in vitro*, PI3K, p-Akt (ser473) and p75NTR expression were significantly increased in tumors derived from Eca109-psb cells compared with that of control cells in control and radiation groups *in vivo* (Figure 7E). However, in the LY294002 and LY294002+radiation groups, no significant difference could be observed in the expression of PI3K, p-Akt (ser473) and p75NTR between the tumors derived from Eca109-psb cells and tumors derived from Eca109-control cells *in vivo* (Figure 7E). What's more, the expressions of PI3K, p-Akt (ser473) and p75NTR are all inhibited by LY294002 (Figure 7E).

Taken together, it could be easily concluded that HPV16 E6-E7 promotes the tumorigenesis and radioresistance in ESCC cells *in vivo*. This effect could be eliminated by intraperitoneally being injected with LY294002. IHC results suggest that the activation of PI3K/Akt signaling pathway induced by HPV16 E6-E7 could be inhibited by LY294002 *in vivo*.

## DISCUSSION

Cancer development is a highly orchestrated process that origins from complex factors, and in ESCC high-risk HPV infection is one of them [31, 32]. Our previous study has proposed that HPV16 infection predicts poor prognosis in ESCC patients [21]. However, no clear mechanism has been reported. The present study firstly aims at finding the influence on cell behavior and potential mechanism after HPV16 E6-E7 infecting the ESCC cells.

It have been proved that cancer recurrence, radio-resistance and chemo-resistant were mostly attributed to the presence of CSCs [33–36]. The specific ability of CSCs is to form spheres *in vitro* under non-adherent culture conditions [22, 37, 38]. Meanwhile, it is well established that p75NTR is one of the most important CSCs markers in ESCC [14, 39], which mainly express in the basal layer of esophageal epithelium [40]. Our previous study also demonstrated that p75NTR positive cells significantly increased in Eca109R-50Gy cells (Eca109 cells achieved by accumulated 50 Gy ionizing radiation with high radioresistance and characteristics of CSCs), compared to Eca109 cells [41, 42]. Basing on this, we sent out to investigate the role of HPV16 E6-E7 in the biological behavior of ESCC cells. Transwell assay in this study found that HPV16 E6-E7 promoted the migration and invasion ability significantly (Figure 1C-1F). The spherogenesis assay performed in this study found that HPV16 E6-E7 also induced spherogenesis in ESCC cells (Figure 2A, 2B). Next, flow cytometry was applied to analysis of p75NTR positive cells, and the results showed that HPV16 E6-E7 induced the stemness in ESCC cells because of the increased ratio of p75NTR positive cells in Eca109-psb, TE-1-psb cells and spheres derived from them (Figure 2C–2F). All of the results above indicate that HPV16 E6-E7 induces CSCs phenotypes in ESCC cells.

One of the important regulatory mechanisms of cell growth is the cell cycle distribution [43]. Cell cycle analysis showed that HPV16 E6-E7 caused an accumulation of cells in G2/M phase with significantly reduction in G0/G1 phase (Figure 2G, 2H). In the cell proliferation analysis, CCK8 cell viability assay (Figure 3A) and colony formation assay (Figure 3B, 3C, Supplementary Table S1) suggested HPV16 E6-E7 promoted chemoresistance and radioresistance in ESCC cells, respectively. Then the cell apoptosis assay performed by flow cytometry analysis revealed that HPV16 E6-E7 increased the anti-apoptotic ability of ESCC cells when treated by ionizing radiation (Figure 3D, 3E). All in all, HPV16 E6-E7 increases the chemoresistance, radioresistance and plays an anti-apoptotic effect in ESCC cells.

As the results described above, the potential mechanism that HPV16 E6-E7 induces CSCs phenotypes in ESCC should be detected. Many signaling pathways are commonly involved in maintaining the CSCs phenotypes [44, 45] and the PI3K/Akt is one of them in the ESCC [26, 46]. Side population cells (SP cells) which could not be stained by Hoechst 33342 dye have been proven to be enriched in CSCs in various tumors and cells [47–49]. The PI3K/Akt regulates the SP cells in ESCC cells and specimens [50]. Akt is the major effector kinases in the downstream of PI3K/Akt pathway, inhibition of PI3K/Akt activity attenuate CSCs metabolism and is critical for CSCs quiescence [19, 20].

So we investigated the PI3K/Akt signaling pathway in ESCC cells with LY294002, PI3K siRNA and Akt siRNA. Western blotting indicated that HPV16 E6-E7 increased PI3K, p-Akt (ser473) and p75NTR expression in ESCC cells (Figure 4A, 4B, Figure 5 and Figure 6). Akt siRNA decreases the p-Akt(ser473) and p75NTR expression in Eca109-psb and TE-1-control, TE-1-psb cells (Figure 6). Immunofluorescence also demonstrated that HPV16 E6-E7 improved PI3K, p-Akt (ser473) and p75NTR expression in ESCC cells and spheres from ESCC cells (Figure 4C and 4D). All the data above show that inhibiting PI3K/Akt signaling pathway decreases the p75NTR expression in Eca109-psb and TE-1-psb cells. Consistently, Tetsuji *et.al* [51] also proved that p75NTR expression was applied for CSCs identification and isolation in ESCC cells. Moreover, the PI3K/Akt/c-Myc Axis was closely associated with CSC-like features is ESCC cells as well as there was significant correlation between strong c-Myc expression and a short overall survival in ESCC patients [30, 50].

The xenograft study found that the proliferation effect significantly promoted by HPV16 E6-E7 in Eca109 cells could be inhibited by LY294002 *in vivo* (Figure 7A, 7B). Furthermore, the IHC found the expression of PI3K, p-Akt (473) and p75NTR in the Eca109-psb cells tissues is definitely higher than control cells tissues in the Control group and Radiation group while no difference could be seen in LY294002 group and LY294002+Radiation group (Figure 7E). This further confirms our hypothesis that HPV16 E6-E7 promotes CSCs phenotypes of ESCC cells which can be partly inhibited by the blocking of PI3K/Akt signaling pathway *in vivo*.

In summary, although many previous studies declare the important role of HPV16 infection in the incidence of ESCC [5, 7], the present study is firstly investigate the potential role and mechanism of HPV16 E6-E7 in the development of ESCC. It is supposed that HPV16 E6-E7 function as a CSCs phenotypes promoter in ESCC cells through activation of PI3K/Akt signaling pathway with the up-regulate of p75NTR, a CSCs marker in ESCC cells. However, it is also indicated that the inhibition of PI3K/Akt signaling pathway reverses the effect which induced by HPV16 E6-E7 in ESCC cells, *in vitro* and *in vivo*. These findings proved that HPV16 may be a potential high risk factor for the ESCC development. Targeting the CSCs in ESCC through the inhibition of PI3K/Akt signaling pathway in HPV16 positive tissues is an available therapeutic.

## MATERIALS AND METHODS

### Cell lines and cancer stem-like cell culture

ESCC cell lines Eca109 and TE-1 were purchased from the Chinese Academy of Sciences Cell Bank (Shanghai, China). The authenticity of cancer cell lines was tested by short tandem repeat profiling. All cells were maintained in Dulbecco's Modified Eagle Medium (DMEM, Gibco, USA) supplemented with 10% fetal bovine serum (FBS, Gibco, USA), 100μg/mL streptomycin (Sigma-Aldrich, USA) and 100U/mL penicillin (Sigma-Aldrich, USA) at 37°C with 5% CO_2_ in a humidified incubator.

Esophageal squamous carcinoma stem-like cells were propagated in the SFM which was composed of DMEM/F12 (DMEM, Gibco, USA), 20ng/ml basic fibroblast growth factor (bFGF; Sigma-Aldrich), 20μl/ml B27 supplement (Life Technologies), and 20ng/ml EGF (Sigma-Aldrich). These cells can form neurosphere-like cell aggregates in 14 days in the sphere formation assay.

### Lenti-virus transfection and confirmation of the transfection

The HPV16 E6-E7 lentiviral vector was synthesized by Shanghai Sunbio Medical Biotechnology CO., Ltd. It was constructed by ligating the HPV16 E6-E7 precursor (789bp) into the EcoR I and Xba I sites of the pLVX-EGFP-T2A-Puro lentivector (8934bp). The lentivector was packaged using pCD/NL-BH*DDD Packaging Plasmid mix (Addgene) and transiently cotransfected into 293T cells to generate recombinant virus particles. After 48h of infection, lentivirus in the supernatant was transduced into esophageal squamous carcinoma cells, Eca109 and TE-1, using 5μg/ml of polybrene (Sigma-Aldrich) at the optimal MOI (multiplicity of infection) of each cell. The control lentiviral vector pLVX-EGFP-T2A-Puro which constructed by nonsense sequences stably expressed green fluorescent protein (GFP), while the HPV16 E6-E7 lentiviral vector stably expressed GFP and HPV16 E6-E7. Stable clones were maintained on 5μg/ml of puromycin (Sigma-Aldrich). Fluorescence intensity of GFP was observed by fluorescence microscope to indicate the lentivirus transfection efficiency. The expression of HPV16 E6-E7 was confirmed by Western blotting.

### Gene silencing by small interfering RNA (siRNA)

Silencing PI3K and Akt expression using siRNA was performed following the manufacturer's instructions. PI3K siRNA 1178, 619 and 856; Akt siRNA 945, 652 and 567; Negative control siRNA and GAPDH siRNA were all synthesised by GenePharma. All the siRNA sequences were listed in Supplementary Table S2. Briefly, at 60%-70% confluence, ESCC cells were transfected with Select siRNA duplexes, or the Negative Control siRNA, or the GAPDH siRNA at 10nM using Lipofectamine 3000® (Invitrogen, Waltham, MA). Following 6h/37°C siRNA incubation, fresh medium was then added for another 60h/37°C incubation.

### *In vitro* migration and invasion assays

Cells (5*10^4^ cells per well) were plated on the top side of polycarbonate transwell filters (for migration assay) or plated on the top side of polycarbonate transwell filter coated with matrigel (for invasion assay) in the top chamber of the 24-Well Cell Invasion Assay (Cell Biolabs, Inc). Cells were suspended in medium without serum, and medium supplemented with 20% serum was used as a chemo-attractant in the bottom chamber. The cells were incubated at 37°C for 24 hours (migration assay) or 48 hours (invasion assay). The nonmigratory or noninvasive cells in the top chambers were removed with cotton swabs. The migrated and invaded cells on the lower membrane surface were fixed in 100% methanol for 3 minutes, air-dried, then stained with crystal violet and counted under a microscope.

### *In vitro* spherogenesis assay

Cell suspensions were then plated into ultra-low attachment 96-well plates (Corning) at a density of 2000 cells per well in SFM. To keep volume of media in the well constant, 20 μL SFM was added every 5 days. At the end of the experiment 10μL of trypan blue dye solution was added to each well to detect dead cells. Sphere growth was monitored for 14 days and the number of spheres was counted on day 14. An average of 6 wells was seeded for each radiation dose.

### Cell viability assay

The exponentially growing cells were plated onto 96-well plates (Corning) in complete DMEM at a density of 6.0*10^3^ cells per well and treated by cisplatin (Sigma-Aldrich, UK; dissolved in 0.9% NaCl,) at different concentration: 2, 5, 10, 20, 50 μmol/L, then incubated at 37°C, 5% CO_2_. After 24, 48 and 72h, the medium was replaced with 100μL DMEM supplemented with 10% FBS and 10μL CCK-8 (Cell counting Kit-8, Beyotime, China) solution and incubated for additional 2h at 37°C. The solution of each sample was aspirated and the absorbance was measured spectrophotometrically at 450 nm. Six parallel replicates of each sample at each time point were prepared during this cell viability assay.

### Flow cytometry

Flow cytometry was performed to detect the ratio of p75NTR^+^ cells in ESCC cells. Briefly, Cells were trypsinized and collected by centrifugation. Then cells were washed and suspended in buffer and incubated with p75NTR-PE antibody (BD Bioscience) at 4°C for 2h. The ratio of p75NTR^+^ cells in each samples were analyzed by FASC Calibur MT flow cytometer (BD Bioscience).

Cell cycle was detected by flow cytometry using a cell cycle analysis kit (BD Bioscience). In brief, at least 1*10^6^ cells were harvested and washed, then cells were fixed in ice-cold 70% ethanol for at least 2h at 4°Cwashed the cells again and stained with a solution containing 50μg/ml PI and 50 RNase at room temperature for 30 min. Cell cycle was analyzed with a FACS Calibur MT flow cytometer (BD Bioscience).

Cell apoptosis was detected by flow cytometry using an Annexin V-APC apoptosis detection kit (BD Bioscience). In brief, cells were harvested and washed, then cells were suspended in binding buffer and incubated with Annexin V-APC and propidium iodide (PI) at 4°C, the cells were double stained with Annexin V-APC and PI according to the manufacturer's instructions. Early apoptosis and the late apoptosis were determined by Annexin V+/PI- staining and Annexin V+/PI+ staining, respectively. The percentage of apoptosis cells in each sample was examined using the FASC Calibur MT flow cytometer (BD Bioscience).

### Colony formation assay

The cells in the monolayer culture were irradiated with graded doses of X-ray by using a linear accelerator at the dose of 0, 2, 4, 6, 8 Gy. The cells that remained attached were removed from the culture flask by exposing to 0.05% trysin solution. Afterward, these cells were re-plated in triplicate onto 6-well plates in complete DMEM to determine the colony-forming ability. After 14d of incubation, the plates were stained with 0.5% crystal violet in absolute methanol, and the colonies consisting of at least 50 cells were recorded. The survival fraction (SF) was calculated by dividing the number of colonies at a certain radiation dose by the product of the number of cells seeded at this dose and the corresponding colony-forming rate. Lines are fitted using the Single hit multi-target model (SF=1-(1-e^−D/D0^)^n^) with GraphPad Prism version 5.01.

### Western blotting

Cells were lysed in RIPA lysis buffer (Pioneer Technology, Xi'an, China) with protease inhibitor cocktail tablets and phosphatase inhibitor cocktail tables (Roche). Then cell lysates were centrifuged at 12,000 r.p.m (revolutions per minute) for 40 min at 4°C. The supernatant was harvested and the protein concentration was determined using a Pierce BCA protein assay kit (Thermo scientific, Fremont, USA). An equivalent amount of protein was separated by SDS-PAGE and transferred to polyvinyldifluoride membranes (Millipore, Billerica, USA). The membranes were probed with the primary antibodies overnight at 4°C, followed by a secondary anti-rabbit or mouse IgG conjugated with HRP. Signals were detected using chemiluminescence reagent (Millipore) and ChemiDoc System (Bio-Rad, Hercules, USA). Primary antibodies were used at a 1:1000 dilution and the antibodies were against: HPV16 E6 (ab70, Abcam, USA), p75NTR (MAB367, R&D, USA), PI3K (#4249, CST, USA), Akt (#9272, CST, USA), p-Akt(ser473) (#4060, CST, USA), GAPDH (#5174, CST, USA), β-actin (#3700, CST, USA) was used as the endogenous reference. Secondary antibodies used were horseradish peroxidase (HRP) - conjugated anti-rabbit (#4414, CST, USA) and anti-mouse (#4410, CST, USA).

### Immunofluorescence

Cells seeded on coverslips were fixed with 4% paraformaldehyde for 30 min after three times PBS washing and permeabilized in PBS with 0.1% TritonX-100. Then cells were blocked with 5% BSA at room temperature for 1 h. Primary antibody incubation was performed overnight at 4°C. Fluorescent secondary antibody was incubated for 1h at room temperature after three times PBS washing and DAPI (#4083, CST, USA) was applied to counterstain nucleus. All the primary antibodies were the same with western blotting. Images were examined and captured under the microscopy (Leica, Heidelberg, Germany).

### Xenograft tumor model and treatment

The animal studies were approved by the Institutional Animal Ethics Committee of The First affiliated hospital of Xi'an Jiao Tong University and experiments were performed in accordance with the Animal Ethics guidelines of The First affiliated hospital of Xi'an Jiao Tong University. Female BALB/c nude mice (four weeks old with body weight approximately 15g) were purchased from the Experimental Animal Center of School of Medicine (Xi'an Jiaotong University) and housed in it. After a week's acclimation, the tumor model was established by subcutaneously injecting 2*10^6^ cells per 200μL, Eca109-control and Eca109-psb cells, in the back of each mouse. The mice were randomly distributed into four groups (N=6):Control, Radiation, LY294002 and LY294002 +Radiation group. The mice in the LY294002 group were intraperitoneally injected with LY294002 at a dose of 5 mg/kg body weight twice a week for five weeks. This dose was administered based on the result of our preliminary experiment, which is 10% of the dose with the median effective concentration of LY294002 when administered alone [29]. The mice treated by radiation alone were immobilized in a customized harness. The back was exposed and the remaining parts of the body were shielded with a thick piece of lead. The tumor area was irradiated by an X-ray linear accelerator with a dose of 2Gy twice a week for five weeks. The mice in the group treated with the combined LY294002 and radiation were irradiated after the injection of LY294002 every time. The control group received a 0.1% DMSO/sodium chloride solution without drugs. Above all of these are operated after tumor cells injection for three weeks when the tumor volume in each group could be macroscopied and measured. The cumulative radiation exposure dose in the radiation group and combined group are 20 Gy. The mice were monitored daily for body weight, behavior and water/food consumption. Tumor growth was monitored by measuring the tumor size in two orthogonal dimensions with Vernier calipers every 4 days; the tumor volume was calculated according to the following formula: length* width^2^*0.5. The mice were anesthetized and sacrificed with eight weeks feeding time. The tumors were surgically excised, weighed, fixed in 10% formalin, embedded in paraffin, and cut into thick slices (4μm) for hematoxylin and eosin staining (H&E) to confirm tumor formation. Inhibition rate was defined as: 1- tumor weight or volume of the treatment group / tumor weight or volume of the control group. The solid tumors were then surgically removed and fixed in 10% formalin neutral buffer solution.

### Immunohistochemistry (IHC)

Immunohistochemical staining was performed according to a standard protocol using a Hypoxyprobe™-1 kit. All the primary antibodies were the same with western blotting and selected at the concentration of 1:100. PBS, instead of primary antibody, was used as a negative control. All of the animal procedures were conducted in accordance with the Guide for the Care and Use of Laboratory Animals of the National Institutes of Health. Experimental protocols were approved by the Animal Care and Use Regulation of Xi'an Jiaotong University. Images were captured using a microscopy (Leica, Heidelberg, Germany) and five random fields of each were chosen from each specimen for quantitative analysis.

### X-ray radiation treatment

Cells were irradiated with 0, 2, 4, 6, 8 Gy of X-ray radiation using the X-ray linear accelerator (Clinac 2100EX, Varian Medical Systems). The treated cells were then used for the experiments as described above.

### Statistical analysis

All results are present as mean ± SD from at least three independent experiments. Statistical analysis was performed using Student's *t*-test and followed by ANOVA analysis in SPSS 16.0. Differences between groups were considered to be statistically significant at *P*<0.05 (*). Graphs were made in GraphPad Prism 5.01 (GraphPad Software, San Diego, California, USA).

## SUPPLEMENTARY MATERIALS TABLES


