# Supplementary material for: HPV16 E6-E7 induces cancer stem-like cells phenotypes in esophageal squamous cell carcinoma through the activation of PI3K/Akt signaling pathway in vitro and in vivo
Source: Oncotarget. 2016 Jul 30;7(35):57050–65. doi: 10.18632/oncotarget.10959 (PMC5302972; doi:10.18632/oncotarget.10959)
Supplement: Supplementary file 1 [file oncotarget-07-57050-s001.pdf]

HPV16 E6-E7 induces cancer stem-like cells phenotypes in esophageal squamous cell carcinoma through the activation of PI3K/Akt signaling pathway *in vitro* and *in vivo*

SUPPLEMENTARY TABLES

Supplementary Table S1: Constant value of radiation biology were calculated and analysed in ESCC cells

	D0	Dq	k	N	SF2
Eca109-control	1.99±0.27	0.15±0.41	0.51±0.07	1.13±0.29	36.97±4.05
Eca109-psb	2.89±0.09**	0.80±0.30	0.35±0.01*	1.32±0.15	61.20±6.01***
TE-1-control	2.31±0.32	0.63±0.60	0.44±0.06	1.38±0.37	51.52±5.38
TE-1-psb	3.84±0.83*	1.52±0.92	0.27±0.05*	1.60±0.44	73.13±4.21***

Constant values of radiation biology were calculated based on the data of colony formation assay. D_0 (D_0 = 1/k) is the mean lethal dose which 63% cells were hitted and causing death. D_q is the quasithreshold dose which represents the shoulder of the dose-survival curve. The higher value of D_0 and D_q represent the more radioresistance of cells. N is extrapolation number of the dose-survival curve which positive related with the repair ability of cells. SF_2 is the survival fraction of cells after expose to 2Gy ionizing radiation and represents the radioresistance of cells. All the data above are represented as mean±S.D. of three independent experiments. **P*<0.05, ***P*<0.01,****P*<0.001.

Supplementary Table S2: The sequences of siRNAs used in this study

siRNA name	Oligonucleotides(5'-3')	
	Sense sequence	Antisense sequence
PI3K-1178	GCAACCUACGUGAAUGUAATT	UUACAUUCACGUAGGUUGCTT
PI3K-619	GGACCUCAAUUCACCUCAUTT	AUGAGGUGAAUUGAGGUCCTT
PI3K-865	GCUAUCCUCUGAACAACUATT	UAGUUGUUCAGAGGAUAGCTT
Akt-945	GCUGAAGAGAUGGAGGUGUTT	ACACCUCCAUCUCUUCAGCTT
Akt-652	GCACCUUCAUUGGCUACAATT	UUGUAGCCAAUGAAGGUGCTT
Akt-567	GCUAUUGUGAAGGAGGGUUTT	AACCCUCCUUCACAAUAGCTT
GAPDH	UGACCUCAACUACAUGGUUTT	AACCAUGUAGUUGAGGUCATT
Negative control	UUCUUCGAACGUGUCACGUTT	ACGUGACACGUUCGGAGAATT
